# Association between ramadan fasting, sleep disturbances, fatigue, and academic performance among Iraqi university students: A cross-sectional study

**DOI:** 10.1186/s12889-025-24482-6

**Published:** 2025-09-24

**Authors:** Heba M. Attash, Omar T. Dawood, Mohammed I. Aladul, Narmin S. Essa

**Affiliations:** 1 College of Pharmacy, University of Mosul, AL-Majmoaa Street, Mosul, Ninevah, 41002 Iraq; 2 College of Pharmacy, Ninevah University, AL-Jawsaq, Mosul, Ninevah, 41002 Iraq; 3 Nineveh Health Directorate, Al-Salam Teaching Hospital, Ministry of Health, Mosul, Ninevah, 41002 Iraq

**Keywords:** Academic performance, Fasting, Iraq, Medical students, Mental fatigue, Physical fatigue, Ramadan, Sleep disturbance

## Abstract

**Background:**

Ramadan fasting intersects with university students’ academic and health behaviors, but its effects on sleep, fatigue, and academic performance remain contested. This study examines these relationships in Iraqi medical students, a population experiencing both academic rigor and cultural observance.

**Methods:**

A cross-sectional web-based survey was conducted among 922 Iraqi medical students from March 26 to April 9, 2024. Validated Arabic versions of the Epworth Sleepiness Scale (ESS-AR; range: 0–24) and the Fatigue Questionnaire (FQ-AR; total score: 0–11, with subscales for physical [0–7] and mental fatigue [0–4]) were administered. ESS-AR scores were interpreted as follows: 0–5 = low sleepiness, 6–10 = normal, 11–12 = mild, 13–15 = moderate, and 16–24 = severe. An FQ-AR total score > 4 indicated severe fatigue. Academic performance was assessed using a pre-tested, culturally adapted questionnaire.

**Results:**

Most students (90.1%) reported fasting-related sleep disruptions. Low academic performance was observed in 61.4% of students, and it was associated with higher levels of physical fatigue (adjusted odds ratio [aOR] = 0.72, *p* < 0.01) and mental fatigue (aOR = 0.60, *p* < 0.01). Counterintuitively, stress (aOR = 1.68, *p* < 0.01) and sleep disturbances (aOR = 2.54, *p* < 0.01) were linked to better performance. This may reflect reverse causality: high-achieving students maintain rigorous study habits during Ramadan, inadvertently increasing their stress and sleep disruptions.

**Conclusions:**

Our findings demonstrate that Ramadan fasting significantly impacts students’ sleep patterns and fatigue levels, with nuanced effects on academic performance. While fatigue negatively correlated with outcomes, the counterintuitive stress-performance association suggests high-achievers may maintain performance despite challenges. Future research should explore targeted interventions to support student wellbeing during religious observances, while institutions should adapt policies to accommodate the unique challenges faced by fasting students.

## Background

University students worldwide face significant physiological and psychological challenges, ranging from academic pressures to disrupted sleep patterns [1, 2]. For Muslim students observing Ramadan, these challenges are compounded by daytime fasting - a practice involving complete abstinence from food and drink from dawn to sunset [3]. While the spiritual benefits of fasting are well-established [4], its relationships with sleep, fatigue, and academic performance remain contested, particularly in high-intensity academic environments like medical schools.

Existing research presents conflicting findings about fasting’s associations. A study by Bahammam (2004) [5] suggested improved sleep quality during Ramadan, while studies by Chennaoui et al. (2009) [6], Nugraha et al. (2017) [7], and Abaidia et al. (2020) [8] demonstrated significant sleep disruptions and daytime fatigue. These discrepancies may arise from variations in study populations, cultural practices, fasting durations, and environmental conditions, as seen in comparative studies. For instance, Qasrawi et al. (2017) [9] reported earlier sleep onset during Ramadan, potentially due to altered melatonin secretion, whereas Faris et al. (2020) [10] found increased daytime sleepiness in their meta-analysis. Similarly, while some evidence links fasting to reduced stress [11, 12], other studies connect it with increased perceived fatigue [7, 13].

These mixed findings take on particular significance for Iraqi medical students, who represent a critical yet understudied population facing four compounding factors: exceptional academic demands requiring 60–70 weekly study hours [14], near-universal cultural observance with 98% of Iraqi Muslims fasting during Ramadan [15], environmental stressors from summer temperatures often exceeding 40 °C (104 °F), and extended daily fasting durations reaching 16 h in summer months.

Despite these stressors, no prior study has simultaneously examined the effects of Ramadan fasting on sleep parameters, fatigue levels, and academic performance in this group. This gap is particularly notable given that medical students’ academic success directly impacts future healthcare quality.

Our hypothesis is that Ramadan fasting will negatively affect sleep patterns and increase fatigue levels, ultimately leading to decreased academic performance among Iraqi medical students. This study investigates the associations between Ramadan fasting, sleep disturbances, fatigue, and academic performance among Iraqi medical students. The findings will provide evidence to help universities develop targeted support strategies during this culturally significant period.

## Methods

### Study design and participants

This cross-sectional study used a web-survey distributed among Iraqi university students in medical colleges, including medicine, pharmacy, dentistry, and nursing, between the 26th of March and the 9th of April 2024. Participants were eligible if they were registered as undergraduate students and observed Ramadan fasting. Students were excluded if they had chronic diseases, a history of sleep disturbances, or used medications or herbal supplements known to affect alertness or sleep, such as benzodiazepines, diuretics, or corticosteroids.

### Data collection

The survey employed a snowball sampling technique where the survey link was distributed by student representatives to their peers through closed academic social media groups. While practical for reaching the target population, this method may introduce selection bias by potentially overrepresenting students who are more academically or socially engaged. Participants received study information, including eligibility criteria and an informed consent notice. The web-based questionnaire was hosted on Microsoft Forms and contained four main sections: demographic characteristics and personal habits; academic performance during Ramadan, assessed using a pre-tested, culturally adapted six-item scale based on Almutairi et al. (2022) [16]; daytime sleepiness, measured by the Arabic Epworth Sleepiness Scale (ESS-AR) [17]; and fatigue, assessed using the Arabic Fatigue Questionnaire (FQ-AR) [18].

The academic performance scale had a total score ranging from 0 to 9, with higher scores indicating better perceived performance. Each of the six items was scored from 0 to 2, where 0 = negative impact, 1 = no impact, and 2 = positive impact of fasting. Items were summed to create a total performance score. For example: “During Ramadan, my ability to concentrate during study sessions has. decreased, no change, or improved “. The ESS-AR is an 8-item scale with a total score ranging from 0 to 24, interpreted as follows: 0–5 = low sleepiness, 6–10 = normal, 11–12 = mild, 13–15 = moderate, and 16–24 = severe [17]. The FQ-AR consists of 11 items (7 for physical fatigue, 4 for mental fatigue), with a total score of 0–11. A total score > 4 indicates severe fatigue symptoms [18]. Breakfast timing was categorized based on typical meal patterns during Ramadan. All instruments were linguistically and culturally adapted for the Iraqi context.

### Validation

Before piloting, face validity was assessed by a panel of experts from different pharmacy colleges in Iraq who evaluated the clarity and relevance of each question. The questionnaire was revised based on their feedback to improve clarity. A second review was conducted by three experts from the Colleges of Pharmacy at the Universities of Baghdad, Mosul, and Al-Noor, who identified some redundant or vague elements. The final version was developed after these revisions.

### Sample size

A sample size calculation was performed using Raosoft online sample size calculator (https://www.raosoft.com) to achieve a 95% confidence level with a 5% margin of error, based on an estimated population of Iraqi university students observing Ramadan fasting. This yielded a minimum required sample size of 378 participants. Considering potential incomplete responses associated with cross-sectional studies and web-survey and to enhance statistical power, a total of 922 responses were collected, exceeding the calculated sample size.

### Statistical analysis

Data were analyzed using IBM SPSS Statistics version 25 (IBM Corp., Armonk, NY, USA). Descriptive statistics, including frequencies, percentages, means, standard deviations, medians, and interquartile ranges, were calculated. The median split method was used to classify the level of study performance [19], whereby participants scoring at or below the actual sample median score of 6 were classified as having ‘Low’ academic performance, and those scoring above 6 were classified as having ‘High’ academic performance. This method was chosen to dichotomize the continuous performance score for logistic regression analysis, facilitating the interpretation of factors associated with relatively better or worse performance within the sample [19]. The assumption of normality for continuous variables (age, ESS-AR score, physical fatigue score, and mental fatigue score) was assessed using both statistical and graphical methods. Statistical evaluation was performed using the Kolmogorov-Smirnov test. Graphical assessment included visual inspection of histograms, Q-Q plots, and boxplots to evaluate the distribution and identify potential deviations from normality. Non-parametric statistical tests were used due to non-normal distributions, with Mann-Whitney U and Kruskal-Wallis tests for group comparisons. Effect size (Cohen’s d) was calculated for all binary group comparisons (Mann-Whitney U tests), and 95% confidence intervals were computed using the formula [20]:

$$\begin{aligned}{SE}_{d}&=\sqrt{\frac{{n}_{1}+{n}_{2}}{{n}_{1}{n}_{2}}+\frac{{d}^{2}}{2\left({n}_{1}+{n}_{2}\right)}}\:,\\& \quad 95\%\:CI=d\pm 1.96\times S{E}_{d}\end{aligned}$$

where, d is the Cohen’s d effect size, SE_d_ is the standard error of d, n_1_ is the sample size of group 1, and n_2_ is the sample size of group 2.

These confidence intervals provide a range within which the true population effect size is likely to lie with 95% confidence, enhancing the interpretability of findings [20]. Effect sizes (Cohen’s d) values of 0.2, 0.5, and 0.8 were interpreted as small, medium, and large effects, respectively [21, 22]. Binary logistic regression evaluated factors affecting study performance using the median split method to classify performance levels. Model fit was assessed using the Hosmer-Lemeshow test. To assess multicollinearity among predictors in the logistic regression model, the Variance Inflation Factor (VIF) was calculated. All VIF values were below 2.0 (range: 1.01–1.75), indicating no significant multicollinearity. Cases with missing values were excluded listwise due to minimal missingness (< 5%).

### Ethical considerations

The study was conducted in accordance with the Declaration of Helsinki and was approved by the Scientific Committee of the Clinical Pharmacy Department, College of Pharmacy, University of Mosul (Approval Code: 11/EECUOM/2024; Date of Approval: 12 February 2024). Informed consent was obtained from all participants.

## Results

The study included 922 university students. No cases were excluded due to missing data, as missingness was less than 5% and managed through listwise deletion as outlined in the Methods. Table 1 illustrates the majority of participants were female (65.8%), with a mean age of 21.8 ± 3.4 years. Participants represented various academic years, from first-year students (29.5%) to sixth-year students (0.7%). Approximately one-fifth of the students (20.7%) were employed in part-time jobs while pursuing their studies.

**Table 1:** Demographic characteristics and ramadan behaviors of Iraqi medical students (*N* = 922)

Variable	*n* (%)
**Gender**	
Female	607 (65.8%)
Male	315 (34.2%)
**Educational Stage**	
First Stage	272 (29.5%)
Second Stage	104 (11.3%)
Third Stage	88 (9.5%)
Fourth Stage	166 (18.0%)
Fifth Stage	286 (31.0%)
Sixth Stage	6 (0.7%)
**Breakfast Habits (All days outside Ramadan)**	
**Do you always have breakfast?**	
Yes	341 (37.0%)
No	237 (25.7%)
Sometimes	344 (37.3%)
**Time of Breakfast**	
Right after waking up	440 (47.7%)
From 9 to 11 AM	300 (32.5%)
After 11 AM	182 (19.7%)
**Do you work?**	
Yes	191 (20.7%)
No	731 (79.3%)
**Fasting Effects**	
**Do you feel headache during fasting?**	
Yes	549 (59.5%)
No	373 (40.5%)
**Do you feel anxiety/stress during fasting?**	
Yes	353 (38.3%)
No	569 (61.7%)
**Does fasting affect your sleep schedule?**	
Yes	831 (90.1%)
No	91 (9.9%)
**Academic Factors**	
**Do you go to college during Ramadan?**	
Yes	891 (96.6%)
No	31 (3.4%)

Concerning breakfast consumption habits outside of Ramadan, about half of the participating students (47.7%) reported typically eating breakfast immediately after waking up, while 32.5% ate between 9 and 11 AM, and 19.7% after 11 AM. During Ramadan, nearly all participants (96.6%) reported attending classes while fasting. More than half (59.5%) of participants experienced headaches during fasting, while 38.3% reported feelings of anxiety or stress. A significant majority (90.1%) indicated that fasting affected their sleep schedule. Regarding academic outcomes, using the median split method (where the actual sample median performance score was 6, as detailed in Methods), 61.4% of students were classified as having low academic performance levels based on the median split method.

Table 2 illustrates the distribution of sleepiness and fatigue scores among the fasting students. The median ESS-AR score was 12.0 (IQR: 9–15), suggesting moderate daytime sleepiness among participants. The median physical fatigue score was 5.0 (IQR: 3–7), while the median mental fatigue score was 2.0 (IQR: 1–3).

**Table 2:** Distribution of sleepiness and fatigue scores among fasting students

Variable	Maximum	Minimum	Mean ± SD	Median	IQR (Q1-Q3)
Epworth Sleepiness Score	24	0	12.4 ± 4.5	12	9–15
Physical Fatigue Score	7	0	4.8 ± 2.1	5	3–7
Mental Fatigue Score	4	0	1.6 ± 1.2	2	1–3

Epworth Sleepiness Score range (0–24), Physical Fatigue Score (0–7), and Mental Fatigue Score (0–4)

Table 3 illustrates that ESS-AR scores varied significantly by gender (*p* < 0.01), with female students reporting higher sleepiness scores (13.19 ± 4.24) compared to males (10.85 ± 4.59), representing a medium effect size (Cohen’s d = 0.53, 95% CI [0.37–0.69]). Although statistically significant, the difference in ESS-AR scores by gender may not be clinically substantial. Female students also showed significantly higher physical fatigue scores (5.09 ± 2.01 vs. 4.11 ± 2.32, *p* < 0.01, Cohen’s d = 0.45, 95% CI [0.29–0.61]) (Table 4). Table 5 illustrates mental fatigue scores across demographic and behavioral factors. A significant difference in mental fatigue was observed across educational stages (*p* = 0.03). Post hoc pairwise comparisons using Dunn’s test with Bonferroni correction revealed that first-year students reported significantly higher mental fatigue compared to fourth-year students (adjusted *p* = 0.04). No other pairwise comparisons between stages reached statistical significance.

**Table 3:** Associations between demographic factors and daytime sleepiness (ESS-AR Scores)

Variable	Mean ± SD	*p*-value	Effect Size (95% CI)
**Gender** ^**†**^		< 0.01*	0.53 (0.37–0.69)
Female (*n* = 607)	13.19 ± 4.24		
Male (*n* = 315)	10.85 ± 4.59		
**Educational stage** ^**††**^		0.64	-
First stage (*n* = 272)	12.59 (4.89)		
Second stage (*n* = 104)	11.75 (4.06)		
Third stage (*n* = 88)	12.39 (4.48)		
Fourth stage (*n* = 166)	12.21 (4.24)		
Fifth stage (*n* = 286)	12.55 (4.43)		
Sixth stage (*n* = 6)	12.33 (4.17)		
**Do you work?** ^**†**^		0.67	0.01 (–0.15–0.17)
Yes (*n* = 191)	12.35 (4.88)		
No (*n* = 731)	12.40 (4.40)		
**Breakfast habits (all days outside Ramadan)**			
**Do you always have breakfast?** ^**††**^		0.39	-
Yes (*n* = 341)	12.66 (4.33)		
No (*n* = 237)	12.06 (4.95)		
Sometimes (*n* = 344)	12.36 (4.33)		
**Time of breakfast** ^**††**^		0.24	-
Right after waking up (*n* = 440)	12.30 (4.31)		
From 9 to 11 A.M. (*n* = 300)	12.73 (4.46)		
After 11 A.M. (*n* = 182)	12.07 (4.98)		
**Fasting Effects**			
Headache during fasting^**†**^		< 0.01*	0.41 (0.25–0.57)
Yes (*n* = 549)	13.12 ± 4.41		
No (*n* = 373)	11.33 ± 4.42		
Stress/anxiety during fasting^**†**^		< 0.01*	0.55 (0.39–0.71)
Yes (*n* = 353)	13.89 ± 4.45		
No (*n* = 569)	11.47 ± 4.28		
Sleep affected by fasting^**†**^		< 0.01*	0.62 (0.41–0.83)
Yes (*n* = 831)	12.66 ± 4.46		
No (*n* = 91)	10.00 ± 4.14		
**Academic Factors**			
College attendance during Ramadan^**†**^		0.02*	0.35 (–0.01–0.71)
Yes (*n* = 891)	12.44 ± 4.50		
No (*n* = 31)	10.96 ± 4.17		

ESS-AR Score range: 0–24 (higher = greater sleepiness), Effect size interpretation: 0.2 = small, 0.5 = medium, 0.8 = large. ^†^Mann-Whitney U tests for binary comparisons. ^††^Kruskal-Wallis for multi-group comparisons. Effect sizes (Cohen’s d) are reported with 95% confidence intervals in brackets

**Table 4:** Physical fatigue scores by demographic and behavioral factors

Variable	Mean ± SD	*P*-value	Effect Size (95% CI)
**Gender** ^**†**^		< 0.01*	0.45 (0.29–0.61)
Female (*n* = 607)	5.09 (2.01)		
Male (*n* = 315)	4.11 (2.32)		
**Educational stage** ^**††**^		0.53	-
First stage (*n* = 272)	4.86 (2.11)		
Second stage (*n* = 104)	4.90 (2.06)		
Third stage (*n* = 88)	4.78 (2.08)		
Fourth stage (*n* = 166)	4.44 (2.30)		
Fifth stage (*n* = 286)	4.79 (2.20)		
Sixth stage (*n* = 6)	4.33 (2.42)		
**Do you work?** ^**†**^		0.21	0.06 (–0.10–0.22)
Yes (*n* = 191)	4.87 (2.25)		
No (*n* = 731)	4.73 (2.14)		
**Breakfast habits (all days outside Ramadan)**			
**Do you always have breakfast?** ^**††**^		0.70	-
Yes (*n* = 341)	4.83 (2.14)		
No (*n* = 237)	4.69 (2.25)		
Sometimes (*n* = 344)	4.73 (2.13)		
**Time of breakfast** ^**††**^		0.45	-
Right after waking up (*n* = 440)	4.75 (2.15)		
From 9 to 11 A.M. (*n* = 300)	4.86 (2.15)		
After 11 A.M. (*n* = 182)	4.59 (2.24)		
**Fasting Effects**			
Headache during fasting^**†**^		< 0.01*	0.93 (0.79–1.07)
Yes (*n* = 549)	5.51 (1.74)		
No (*n* = 373)	3.65 (2.27)		
Stress/anxiety during fasting^**†**^		< 0.01*	1.01 (0.86–1.16)
Yes (*n* = 353)	5.90 (1.50)		
No (*n* = 569)	4.05 (2.21)		
Sleep affected by fasting^**†**^		< 0.01*	0.88 (0.68–1.08)
Yes (*n* = 831)	4.95 (2.06)		
No (*n* = 91)	3.02 (2.38)		
**Academic Factors**			
College attendance during Ramadan^**†**^		0.46	0.08 (–0.28–0.44)
Yes (*n* = 891)	4.76 (2.17)		
No (*n* = 31)	4.58 (2.06)		

Physical Fatigue Score range: 0–7 (higher = greater fatigue), Effect size interpretation: 0.2 = small, 0.5 = medium, 0.8 = large. ^†^Mann-Whitney U tests for binary comparisons. ^††^Kruskal-Wallis for multi-group comparisons. Effect sizes (Cohen’s d) are reported with 95% confidence intervals in brackets

**Table 5:** Mental fatigue scores by demographic and behavioral factors

Variable	Mean ± SD	*p*-value	Effect Size (95% CI)	Post Hoc (Dunn’s Test, Adjusted *p*)
**Gender** ^**†**^		0.06	0.13 (–0.05–0.31)	-
Female (*n* = 607)	1.66 ± 1.21			
Male (*n* = 315)	1.50 ± 1.22			
**Educational stage** ^**††**^		0.03*	-	
First stage (*n* = 272)	1.73 ± 1.24*			vs. Fourth: *p* = 0.04* vs. all other stages *p* = 1.00
Second stage (*n* = 104)	1.49 ± 1.23			vs. all other stages *p* = 1.00
Third stage (*n* = 88)	1.77 ± 1.16			vs. fourth stage *p* = 0.15 vs. all other stages *p* = 1.00
Fourth stage (*n* = 166)	1.37 ± 1.24			vs. First: *p* = 0.04* vs. third 0.15 vs. fifth = 0.36 vs. all other stages *p* = 1.00
Fifth stage (*n* = 286)	1.63 ± 1.18			vs. fourth *p* = 0.36 vs. all other stages *p* = 1.00
Sixth stage (*n* = 6)	1.16 ± 1.32			vs. all other stages *p* = 1.00
**Do you work?** ^**†**^		0.10	0.14 (–0.04–0.32)	-
Yes (*n* = 191)	1.74 (1.30)			
No (*n* = 731)	1.57 (1.19)			
**Breakfast habits (all days outside Ramadan)**				
**Do you always have breakfast?** ^**††**^		0.58	-	-
Yes (*n* = 341)	1.65 (1.20)			
No (*n* = 237)	1.59 (1.26)			
Sometimes (*n* = 344)	1.57 (1.21)			
**Time of breakfast** ^**††**^		0.68	-	-
Right after waking up (*n* = 440)	1.62 (1.23)			
From 9 to 11 A.M. (*n* = 300)	1.62 (1.17)			
**Fasting Effects**				
Headache during fasting^**†**^		< 0.01*	0.69 (0.53–0.85)	-
Yes (*n* = 549)	1.93 ± 1.14			
No (*n* = 373)	1.13 ± 1.16			
Stress/anxiety during fasting^**†**^		< 0.01*	0.98 (0.83–1.13)	-
Yes (*n* = 353)	2.26 ± 1.08			
No (*n* = 569)	1.20 ± 1.11			
Sleep affected by fasting^**†**^		< 0.01*	0.61 (0.40–0.82)	-
Yes (*n* = 831)	1.67 ± 1.21			
No (*n* = 91)	0.97 ± 1.07			
**Academic Factors**				
College attendance during Ramadan^**†**^		0.21	0.15 (–0.21–0.51)	-
Yes (*n* = 891)	1.62 (1.22)			
No (*n* = 31)	1.44 (1.15)			

Mental Fatigue Score range: 0–4 (higher = greater fatigue), Effect sizes shown for significant comparisons (d = 0.2 small, 0.5 medium, 0.8 large). ^†^Mann-Whitney U tests for binary comparisons. ^††^Kruskal-Wallis for multi-group comparisons. Post hoc Dunn’s test with Bonferroni adjustment revealed a significant difference in mental fatigue between first-year and fourth-year students (adjusted *p* = 0.04). Effect sizes (Cohen’s d) are reported with 95% confidence intervals in brackets

Tables 3, 4 and 5 present comprehensive analyses of factors associated with sleepiness and fatigue. Students who attended classes during fasting reported statistically significant higher mean scores of sleepiness (12.44 ± 4.50) compared to those who did not attend (10.96 ± 4.17, *p* = 0.02, Cohen’s d = 0.35, 95% CI [−0.01–0.71]). Participants experiencing headaches during fasting showed elevated ESS-AR scores (13.12 ± 4.41 vs. 11.33 ± 4.42, *p* < 0.01, Cohen’s d = 0.41, 95% CI [0.25–0.57]), higher physical fatigue scores (5.51 ± 1.74 vs. 3.65 ± 2.27, *p* < 0.01, Cohen’s d = 0.93, 95% CI [0.79–1.07]), and increased mental fatigue scores (1.93 ± 1.14 vs. 1.13 ± 1.16, *p* < 0.01, Cohen’s d = 0.69, 95% CI [0.53–0.85]) compared to those without headaches. Notably, participants reporting anxiety/stress during fasting also demonstrated significantly higher levels of sleepiness (13.89 ± 4.45 vs. 11.47 ± 4.28, *p* < 0.01, Cohen’s d = 0.55, 95% CI [0.39–0.71]) and fatigue (Physical: 5.90 ± 1.50 vs. 4.05 ± 2.21, *p* < 0.01, Cohen’s d = 1.01, 95% CI [0.86–1.16]; Mental: 2.26 ± 1.08 vs. 1.20 ± 1.11, *p* < 0.01, Cohen’s d = 0.98, 95% CI [0.83–1.13]) compared to those without stress/anxiety. Similar patterns were observed for students, indicating fasting affected their sleep schedule.

To further explore the predictors of academic performance, a binary logistic regression model was applied (Table 6). The model demonstrated good fit based on the Hosmer-Lemeshow test (χ²(8) = 5.82, *p* = 0.67) and explained a substantial proportion of variance in academic performance, with a Nagelkerke R² of 0.395, indicating a moderately strong model. Higher levels of physical fatigue (adjusted odds ratio [aOR] = 0.72, 95% CI [0.65–0.80], *p* < 0.01) and mental fatigue (aOR = 0.60, 95% CI [0.52–0.70], *p* < 0.01) were strongly negatively associated with high academic performance. Contrary to initial expectations and highlighting a key counterintuitive finding, stress or anxiety during fasting (aOR = 1.68, 95% CI [1.14–2.48], *p* < 0.01) and disrupted sleep schedules (aOR = 2.54, 95% CI [1.48–4.36], *p* < 0.01) emerged as factors positively associated with high academic performance, suggesting these may be markers of increased academic effort rather than hindrances, possibly due to high-achievers’ commitment. Breakfast timing also showed significant associations, with students who typically ate breakfast between 9 and 11 AM (aOR = 1.58, 95% CI [1.07–2.33], *p* = 0.02) or after 11 AM (aOR = 1.79, 95% CI [1.08–2.96], *p* = 0.03) having higher odds of high academic performance compared to those who ate immediately after waking up.

**Table 6:** Multivariable logistic regression of factors associated with academic performance during ramadan

Variable	aOR (95% CI)	*p*-value	VIF
**Demographic Factors**			
Gender (Female vs. Male)	0.75 (0.52–1.09)	0.14	1.23
Age (per year increase)	1.03 (0.98–1.08)	0.25	1.14
Employed (Yes vs. No)	0.92 (0.63–1.34)	0.71	1.22
College attendance (Yes vs. No)	0.85 (0.35–2.05)	0.72	1.01
**Health Factors**			
Headache during fasting (Yes vs. No)	1.22 (0.85–1.75)	0.28	1.10
Stress/anxiety during fasting (Yes vs. No)	1.68 (1.14–2.48)	< 0.01*	1.34
Fasting affects sleep (Yes vs. No)	2.54 (1.48–4.36)	< 0.01*	1.23
**Fatigue Measures**			
Physical fatigue score (per unit increase)	0.72 (0.65–0.80)	< 0.01*	1.75
Mental fatigue score (per unit increase)	0.60 (0.52–0.70)	< 0.01*	1.58
ESS-AR score (per unit increase)	0.98 (0.94–1.03)	0.37	1.23
**Behavioral Factors**			
Having breakfast in all days other than Ramadan			
Yes, vs. No	1.34 (0.81–2.20)	0.24	1.76
Sometimes vs. No	1.15 (0.78–1.71)	0.47	1.43
Breakfast timing:			
9–11 AM vs. immediate	0.63 (0.42–0.93)	0.02*	1.28
After 11 AM vs. immediate	0.56 (0.33–0.93)	0.03*	1.56

Academic performance was classified based on actual sample median score of 6, as having ‘Low’ academic performance, and those scoring above 6 were classified as having ‘High’ academic performance. aOR = adjusted odds ratio. (*) indicates statistical significance (*p* < 0.05). ESS-AR = Epworth Sleepiness Scale-Arabic version. Model Statistics: Omnibus Test: χ²(14) = 317.09, *p* < 0.01. Hosmer-Lemeshow: χ²(8) = 5.82, *p* = 0.67. Nagelkerke R² = 0.395. Multicollinearity was assessed using Variance Inflation Factors (VIF). All VIF values ranged from 1.01 to 1.75, indicating no significant multicollinearity among predictors

## Discussion

This study examined the relationships between Ramadan fasting and sleep patterns, fatigue levels, and academic performance among Iraqi university students. Our findings demonstrate significant associations between fasting and these key outcomes, with several notable patterns emerging.

The finding that 90.1% of participants reported fasting-related sleep disturbances highlights the substantial impact of Ramadan observance on sleep schedules in this population. This aligns with previous studies reporting increased sleepiness during Ramadan [10, 23]. This sleep disruption likely stems from multiple factors, including altered sleep schedules due to nighttime prayers (Taraweeh) and pre-dawn meals (Suhoor), which can desynchronize circadian rhythms [24, 25]. The high prevalence observed reinforces the need for institutional awareness of these physiological correlates.

Exploring the demographic characteristics further, several factors may influence the observed associations. While female students reported higher levels of sleepiness and physical fatigue, the overall impact of Ramadan fasting on performance appeared consistent across genders. The academic stage of participants ranged widely, but its specific moderating effect on our outcomes warrants further investigation. Additionally, the 20.7% of students engaged in part-time work represent a subgroup potentially facing compounded stressors, which could interact with the effects of fasting. The social dynamics inherent in our sampling method may also have selected for a cohort more accustomed to managing high demands, potentially influencing the nuanced relationship between stress and academic performance observed in this study.

Our most counterintuitive finding—that stress/anxiety and sleep disturbances were associated with better academic performance—contrasts with some studies [26, 27] but aligns with the ‘stressful success’ phenomenon observed among high-achieving students [28, 29]. In this context, reported stress/anxiety may reflect eustress—a positive form of stress linked to high engagement and conscientiousness—rather than purely negative psychological distress. Students may leverage Ramadan’s structured daily routine to optimize study habits [30]. Additionally, religious practices such as fasting may enhance self-discipline and persistence [31, 32], indirectly supporting academic focus despite physiological strain. This suggests that stress during Ramadan may function less as a direct impediment and more as a marker of goal-directed behavior and increased academic effort. However, methodological factors (such as potential self-report bias) may also influence these associations. Future mixed-methods studies are needed to explore the bidirectional relationships and how cultural or religious contexts shape these patterns.

The robust negative associations between fatigue and academic performance (physical fatigue aOR = 0.72; mental fatigue aOR = 0.60) corroborate global evidence on cognitive correlates of fatigue. This is particularly relevant for medical students, whose training demands peak cognitive function. The observed benefit of later breakfast timing (9–11 AM: aOR = 1.58; after 11 AM: aOR = 1.79) suggests that meal scheduling may relate to better academic adaptation during fasting, supporting Adolphus et al.‘s [33] findings. These results have practical implications for educational institutions: identifying students experiencing high fatigue levels could help target support services, and considering flexible scheduling or hybrid learning options during intensive religious observances like Ramadan might help mitigate performance decrements. While this study focuses on Ramadan fasting, these results may also be relevant to broader issues of food insecurity, which affects a growing population of students in developing countries.

### Limitations

While providing valuable insights, this study has several limitations. Its cross-sectional design precludes causal inferences, and self-reported data may introduce bias. Although the snowball sampling method was practical for data collection, it may have introduced selection bias by overrepresenting academically engaged or socially connected students, which could limit the generalizability of the findings to the broader student population and making calculation of a response rate unfeasible. Additionally, the reliance on self-reported academic performance, rather than objective measures like GPA, represents another limitation. Future longitudinal studies using objective measures (e.g., actigraphy for sleep, GPA tracking) would strengthen the evidence base and allow for more robust conclusions about the dynamic relationships between fasting, fatigue, sleep, and academic outcomes.

## Conclusion

This study reveals significant associations between Ramadan fasting and sleep disturbances, fatigue, and academic performance in Iraqi medical students. The paradoxical relationship between stress and academic performance may reflect compensatory efforts by high-achieving students who maintain rigorous study habits despite increased psychological strain. These findings suggest that universities should consider implementing tailored support systems during Ramadan, including flexible class schedules, awareness programs on sleep hygiene, and access to psychological counseling for at-risk students. Institutional adaptations, such as monitoring fatigue levels to identify struggling students and potentially adjusting exam schedules or offering remote learning options during peak fasting periods, can help mitigate the challenges associated with fasting while promoting both academic success and student wellbeing.

## Data Availability

The datasets generated and/or analyzed during the current study are available from the corresponding author upon reasonable request.
